# Direction-Finding Study of a 1.7 mm Diameter Towed Hydrophone Array Based on UWFBG

**DOI:** 10.3390/s24134300

**Published:** 2024-07-02

**Authors:** Su Wu, Junbin Huang, Yandong Pang, Jiabei Wang, Hongcan Gu

**Affiliations:** 1 Department of Weaponry Engineering, Naval University of Engineering, Wuhan 430033, China; 2 Department of Electronic Engineering, Naval University of Engineering, Wuhan 430033, China

**Keywords:** fiber-optic sensor, UWFBG, distributed sensor, hydrophone, direction finding

## Abstract

This paper investigates a 1.7 mm diameter ultra-weak fiber Bragg grating (UWFBG) hydrophone towed array cable for acoustic direction finding. The mechanism of the underwater acoustic waves received by this integrated-coating sensitizing optical cable is deduced, and it is shown that the amplitude of its response varies with the direction of the sound wave. An anechoic pool experiment is carried out to test the performance of such a hydrophone array. The test array is a selection of six sensing fibers, each of which is coiled into 9 cm diameter fiber ring suspended in the water to receive acoustic signals. An average sensitivity of −141.2 dB re rad/μPa at frequencies from 2.5 kHz to 6.3 kHz was achieved, validating the detection of the azimuth of underwater acoustic waves. The ultra-thin towing cable system, with free structure, high sensitivity, and underwater target-detection capability has demonstrated great potential for future unmanned underwater vehicle (UUV) applications.

## 1. Introduction

Fiber-optic hydrophone towed arrays are the key devices that accomplish the tasks of detecting, tracking, localizing, and identifying underwater targets, which arrange hydrophones in a linear array and tow them behind a platform [1,2,3,4,5,6,7]. A key challenge in the realization of a towed array is the difficulty of multiplexing a large number of fiber-optic hydrophones (FOHs) in a linear towed array, while maintaining consistent performances in a thin cable. With the increasing requirements for the technical application of a towed array loaded on a small underwater platform, such as an Unmanned Underwater Vehicle (UUV) and an Autonomous Underwater Vehicle (AUV), the development of thin FOH towed array has great potential to become the next generation of acoustic sensing systems with high integration and on a large scale, which is of great significance for mounting on small underwater platforms [8,9,10].

Generally, FOH towed arrays are based on three kinds of sensor configurations. The conventional structure is complicated with many discrete optical component fusion joints, which make the array not only costly but also difficult to manufacture with poor reliability [11,12,13]. In recent years, demonstrations of a towed array based on fiber coil with a flexible elastic bar have been reported [14,15,16,17,18,19]. F. A. Bruno [20] proposed a mandrel-based optical fiber hydrophone, whose composite mandrel with a plastic shell filled with a compliant solid core and oil achieved a bandwidth of up to 10 k Hz in an FOH array, with an acoustic pressure sensitivity of −136 dB re rad/μPa. Since the mandrel substrate limits the bending radius of the towed array, it requires a large amount of space for UUV storage, leading to difficulties in retracting and releasing the towed hydrophone array, which also affects platform navigation. However, in place of the two previous sensor configurations, distributed acoustic sensing (DAS) technology has been applied to downhole oil and gas exploration [21,22], geological monitoring [23], pipeline safety [24], and perimeter security [25,26]. 

Compared to traditional fiber-optic hydrophones, DAS technology for underwater acoustic sensing applications generally has a lower SNR [27]. In recent years, a great deal of work has been done on the suppression of laser noise, clock asynchrony noise, and polarization fading noise of the coherent system [28], and a series of backscattering enhanced points is employed as the sensing fiber (e.g., UWFBG) to suppress the coherent fading noise [29,30]. There has been a gradual improvement in the SNR of DAS systems used in underwater acoustic sensing. In 2021, Lu [31] presented a DAS system based on coherent detection. In order to increase the sensitivity, an optical sensor cable was prepared with a mandrel winding, the diameter of which was 45 mm. In a lake trial, the acoustic pressure sensitivity reached −146 dB (re rad/μPa), demostrating underwater sound source localization. The authors of [32] demostrated an ultra-highly sensitive distributed hydroacoustic sensing system based on the acoustic sensitization layer and spiral packaged backscattering enhanced optical fiber (BEOF), which reached up to −137.2 dB (re rad/μPa·m). The diameter of the FOH cable was still 22 mm. However, the mandrel substrate limited the diameter and the bending radius of the towed array. As the scale of FOH grows, it is difficult to meet the requirements for the adaptability of small unmanned platforms, such as UUVs. 

In order to reduce the weight and size parameters while easily multiplexing distributed sensors, the researchers present a linear array with a sensitive coating for the fiber instead of a mandrel structure. For example, the Russian six-element hydrophone [17] focused on investigating the sensitization effect of the ‘RTV66’ material coating. Domestic research has mostly focused on fiber-optic coating sensitization [33], signal crosstalk [34], and signal demodulation [35,36] without underwater acoustic direction estimation. However, it has not been reported whether this freely distributed linear array can achieve underwater acoustic direction estimation.

This paper presents the acoustic response and azimuth estimation of underwater sensing using a 1.7 mm diameter UWFBG hydrophone towed array. We investigate the mechanism of the underwater acoustic waves received by a distributed linear array without any mandrel structure; it can be inferred that the amplitude of its element response varies with the direction of the sound wave. In anechoic pool tests, the testing fiber is wound into six fiber-optic rings (six-element hydrophones) are arranged at equal intervals of 0.18 m and suspended in the anechoic pool. The average acoustic pressure sensitivity of the linear array is tested to be −141.2 dB (re rad/μPa) within a frequency range of 2.5 kHz–6.3 kHz. Finally, by adjusting the orientation of the hydrophone array at equal intervals of 15°, the azimuth corresponding to the maximum beam response coincides with the acoustic direction.

## 2. Principle and Simulation

### 2.1. Distributed Acoustic Sensing System

**Figure 1:**
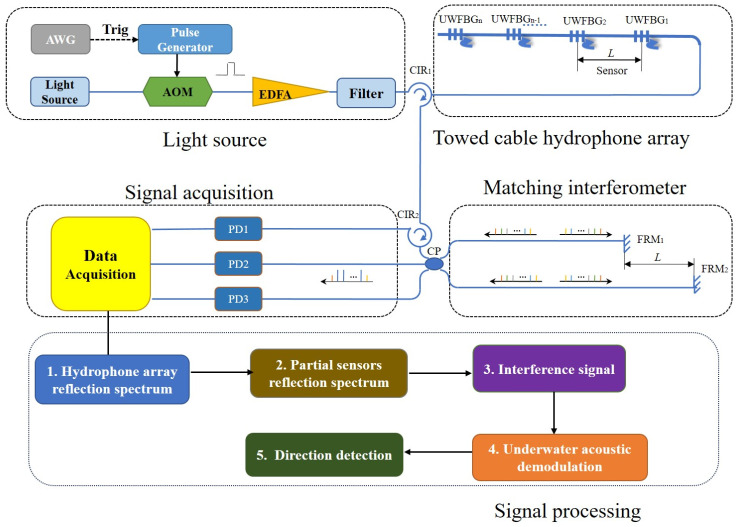
The schematic diagram of the sensing system of the towed cable hydrophone array.

The arrangement of the hydrophone towed array sensing system is shown in Figure 1. 

Firstly, a brief introduction to its working principle is given. The continuous-wave light from an ultra-narrow linewidth laser (ULL) is modulated by the acousto-optic modulator (AOM) to generate laser pulses, which are amplified by an Erbium-doped fiber amplifier (EDFA) and a filter before being injected into a sensing fiber through a circulator. Then the laser pulse enters UWFBGs, which are used as ‘mirrors’, to reflect signals in adjacent UWFBGs, which are matched with the optical path difference *L* of the unbalanced interferometer to realize two-beam interference, and finally it enters the photoelectric detectors (PDs). Since the sensing unit is the fiber, the interference signals contain not only the information of the corresponding UWFBGs in the fiber but also the phase changes within the sensing unit for demodulation, corresponding to the dynamic strains or acoustic waves.

The acoustic signal is captured at a series of the sensing unit, and the amplitude response of the distributed equivalent array elements is related to the angle (α) between the plane wave and the normal of the sensor.

When α = 0, since the optical path difference of the light reflections between two adjacent UWFBGs is zero, the phase of the interference signal can be expressed as

(1) $$\varphi =\frac{4\pi n_{e_{ff}}L}{\lambda_{L_{i}ght}}$$

where *λ_Light_* is the wavelength of the light pulse. When the acoustic pressure acts on the fiber, the phase difference variation is affected by a physical length variation Δ*l* and the refractive index of the fiber Δ*n_eff_* due to the elastic deformation and the photoelectric effect. It can be expressed as

(2) $$\Delta \varphi =\varphi =\frac{4\pi n_{e_{ff}}L}{\lambda_{L_{i}ght}}\;(\frac{\Delta n_{eff}}{n_{eff}}+\frac{\Delta L}{L})$$

### 2.2. UWFBG Theory of Direction Detection for Hydrophones

Different from the acoustic direction detection of the traditional hydrophone, the mechanism of the underwater acoustic waves received by the distributed linear array without any mandrel structure is investigated in this paper. Under far-field conditions, the acoustic waves emitted by the transducer are considered to be plane waves. When they are transmitted to a distributed sensing fiber, the sound pressure acting on the fiber can be expressed as

(3) $$P=P_{0}\cos(\omega t-kz+\varphi_{0})$$

where the *t* is acoustic wave transmission time, *z* is acoustic wave transmission distance, $P_{0}$, ω, $k,z,\varphi_{0}$ representing the initial amplitude, angular frequency, wavenumber, and initial phase of acoustic wave.

**Figure 2:**
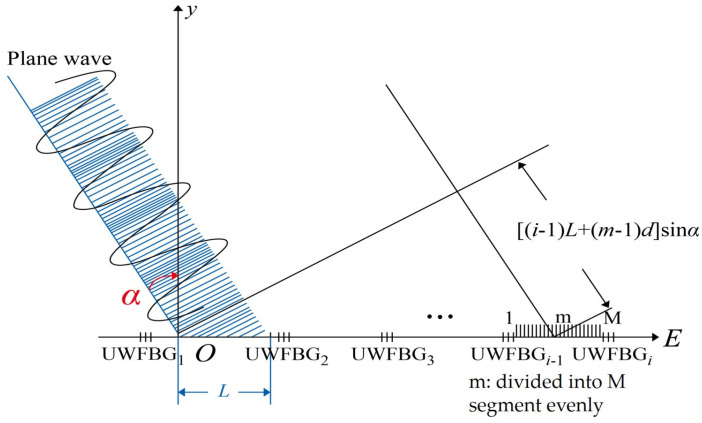
Far-field acoustic waves acting on UWFBG hydrophones.

When far-field plane acoustic waves are incident on the sensing unit in the array, which is shown as in Figure 2, where α is the angle between the plane wave and the normal of the hydrophone. 

When α≠0, the sound pressure received by different parts of the sensing fiber is uneven. The acoustic pressure on each segment of fiber optic of the adjacent UWFBGs is unequal. Based on segmentation theory of the FBG transport matrix, we set up the hydroacoustic effect model. If the *i*^th^ unit is evenly divided into *m* segments (*n* = 1, 2, 3, …, *M*) with a length of *d*, which receive approximately the same acoustic pressure. 

Taking the first segment in the first unit fiber optic as the reference point *O*, the *m*^st^ segment in the *i*^st^ unit fiber optic as the reference point *B* (the length of fiber Bragg gratings is neglected), these acoustic pressures are expressed as

(4) $$P=P_{0}cos(\omega t+\varphi_{0})$$

(5) $$P_{B}=P_{0}cos\{\omega t-k[(m-1)d+(i-1)L]sin\alpha +\varphi_{0}\}$$

Assuming that the sensing fiber is a uniform cylinder, according to the relationship between *n_eff_* and *L* with acoustic pressure [37], Equation (2) can be expressed as

(6) $$\Delta \varphi =LP[-\frac{4\pi n_{e_{ff}}(1-2v)}{\lambda_{L_{i}ght}E}+\frac{2\pi n_{e_{ff}}^{3}(1-2v)(p_{11}+2p_{12})}{\lambda_{L_{ight}}E}]$$

Therefore, by Equations (5) and (6), we have

(7) $$\Delta \varphi_{im}=\left[-\frac{4\pi n_{e_{ff}}\left(1-2v\right)}{\lambda_{L_{i}ght}E}+\frac{2\pi n_{e_{ff}}^{3}\left(1-2v\right)\left(p_{11}+2p_{12}\right)}{\lambda_{L_{ight}}E}\right]\times dP_{0}cos\{\omega t-k[(m-1)d+(i-1)L]sin\alpha +\varphi_{0}\}$$

where the $p_{11}$ and $p_{12}$ are the photo-elastic coefficients of optical fiber, *E* and *v* are the Young’s modulus and Poisson’s ratio, respectively.

Thus, according to the superposition theorem, the phase difference variation of the *i*^th^ unit fiber optic Δ*φ_i_* is expressed as

(8) $$\Delta \varphi_{i}=\sum_{m=1}^{M}\Delta \varphi_{im}$$

Therefore, by Equation (8), we have

(9) $$\;\;\Delta \varphi_{i}=P_{0}d\frac{sin(M\phi ∕2)}{sin(\phi ∕2)}cos[wt+\varphi_{0}-k(i-1)L\;sin\alpha -\frac{k(M-1)dsin\alpha }{2}]$$

where $\phi =kdsin\alpha =\frac{2\pi dsin\alpha }{\lambda }$, when  d→0,  M→∞,

(10) $$\Delta \varphi_{i}=P_{0}L\frac{sin(\frac{\pi Lsin\alpha }{\lambda })}{\frac{\pi Lsin\alpha }{\lambda }}\;cos[wt+\varphi_{0}-(i-1)(\frac{2\pi L\sin\alpha }{\lambda })-\frac{\pi L\sin\alpha }{\lambda }]$$

By Equation (10), the phase difference is regarded as the acoustic pressure response at the equivalent center of the unit fiber optic, which we called the hydrophone element. The signals on the *i*^th^ and (*i* + 1)^th^ hydrophone element are consistent with those of the discrete elements of the linear array, which are expressed by

(11) $$\Delta \varphi =\frac{2\pi L\sin\alpha }{\lambda }$$

Therefore, the beam formation of the continuous linear array is akin to that of a discrete linear array, but the amplitude of its hydrophone response varies with the direction of the acoustic signal. 

**Figure 3:**
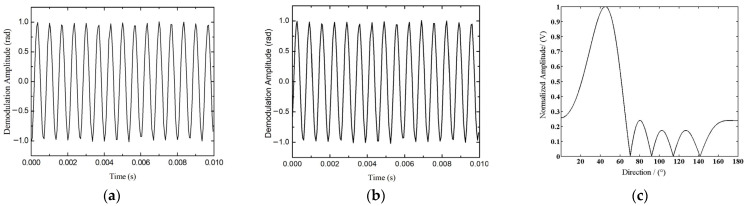
Hydrophone towed cable configuration. (**a**) the *i*^th^ time-domain signal, (**b**) the (*i* + 1)^th^ time-domain signal, and (**c**) azimuth detection.

Under the condition that the transmitting distance *z* and the initial phase *φ* are 0, the phase signals of the interferometric optical pulse are simulated for the two sensors. To achieve spatial sampling and prevent azimuth ambiguity, the horizontal spacing between a pair of UWFBGs is typically less than half of the wavelength. When the acoustic source frequency is *f* = 1500 Hz, the propagation speed of acoustic waves in water is *c* = 1500 m/s. To overcome the influence of the fiber parameters on the results, the amplitude is normalized, and the time domain results are shown in Figure 3a,b. The output amplitude is the maximum value indicated as vertical axis in Figure 3c, which is consistent with the preset values.

It is confirmed by the theoretical analysis and simulation results that the phase relationship of the signal is equivalent to that of a point sensor of fiber-optic hydrophone, although the amplitude of the hydrophone response varies with the direction of the acoustic waves in the distributed UWFBG fiber-optic hydrophone array. Thus, the distributed WFBG hydrophone towed array can form the spatial directivity.

## 3. Experimental Setup and Results

### 3.1. UWFBG Hydrophone Array Design, Fabrication

**Figure 4:**
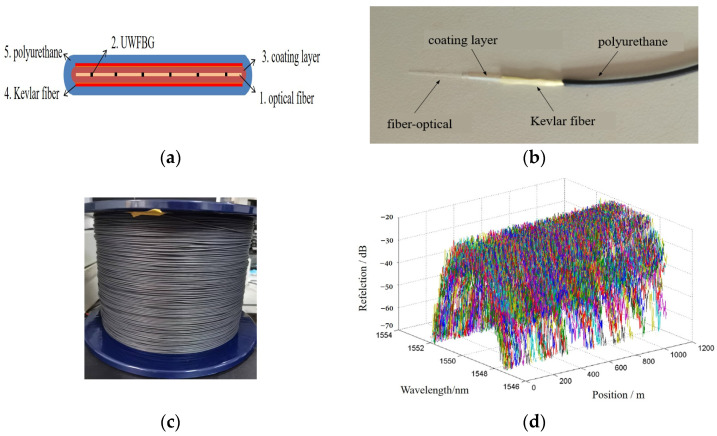
UWFBG hydrophone array configuration. (**a**) structure diagram; (**b**) physical picture; (**c**) detail picture; (**d**) reflected spectra of WFBG hydrophone array.

Figure 4 shows the designed UWFBG hydrophone array schematic diagram in this article. A distributed towed array consisting of 96 UWFBGs spaced 5 m apart, center wavelength of 1550.5 nm, reflectivity of 0.01%, and 3 dB bandwidth of 3 nm. The integrated cabling process is adopted for ultra-thin towed array to make the performance of high sensing and tensile strength, whose structure is shown in Figure 4a, containing an optical-fiber layer with UWFBG, coating layer, and polymer cladding, which are mixed with a Kevlar fiber-reinforced polyurethane sheath. 

Based on our previous work [37], the higher the Poisson’s ratio and the lower the Young’s modulus of the coating layer, the higher the acoustic pressure sensitivity that can be obtained. There is an optimum thickness for the coating, whereas if it is too thick, there is relatively little effect on the sensitivity. Considering the common materials, it is shown in Figure 4b,c that the diameter of the optical cable is 1.7 mm, including the high-density polyethylene coating an optical cable, with a diameter of 0.4 mm, and the polyurethane sheath with a thickness of 0.55 mm to improve the mechanical strength and sensitivity.

In order to accurately analyze the consistency of the UWFBG hydrophone array, we have tested the reflected spectra of the UWFBG arrays with a LG1-100B backlight reflectometer; the result is shown in Figure 4d. It can be clearly seen that the reflectivity is −40 dB and the 3 dB bandwidth is 3 nm. The center wavelength of the UWFBGs is 1550.5 nm, which matches the center wavelength of the light source.

**Figure 5:**
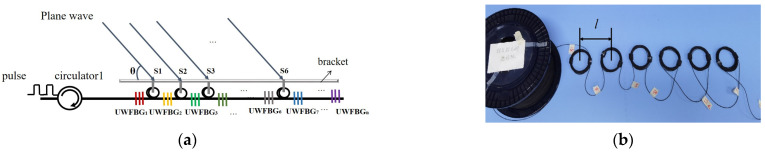
Six-elements testing array: (**a**) wiring diagram; (**b**) fiber-optic rings.

Due to the limited size of the pool and its high-frequency acoustic absorption, the array with a length of 5 m for each element cannot be placed in a straight line. To overcome these difficulties, we fabricated a testing array. The testing fiber was wound into six fiber-optic rings (six-element hydrophones) arranged at equal intervals of 0.18 m, suspended in the anechoic pool, as shown in Figure 5a.

The center points of the ring are fixed at S_1_, S_2_, S_3_, S_4_, S_5,_ and S_6_. Since the center point of the optical fiber ring is approximately the geometric center of the 5 m sensing fiber, according to the principles of acoustics, each geometric center point can be regarded as the equivalent acoustic center of distributed sensors. Therefore, it is flexible to adjust the frequency range of underwater acoustic detection by changing the length of the distance l. In this experiment, the center frequency of the underwater acoustic signal is set to 4 kHz. According to the principle of underwater acoustic detection, l is set to 0.18 m, which is less than half of the acoustic wavelength. In Figure 5a, the angle between the plane wave and the axis of the array is recorded as θ (where θ + α = π/2). Finally, the azimuth of the acoustic wave can be estimated from the acoustic pressure signals of the array.

### 3.2. Experimental Setup

**Figure 6:**
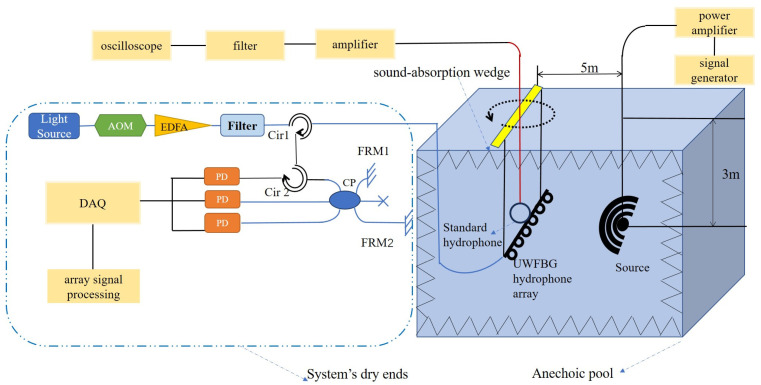
Schematic of the experimental setup.

The experimental setup of the 6-element UWFBG hydrophone array with a simple manual swivel table is shown in Figure 6. The dry ends called the demodulation system are in the dash line of the schematic diagram. The UWFBG hydrophone array and the transducer are placed under 3 m of the anechoic pool.

In the demodulation system, the light source (DFB-M-1550-200-F-10-09MPF-FC/APC) is a ULL laser with a linewidth of 200 kHz and a center wavelength of 1550 nm to match that of the UWFBGs; output power is 10 mW. The light is injected into the AOM (SGTF100-1550-1T) to produce a 20 ns pulse train to ensure that the lap time between two reflected pulses from two adjacent UWFBGs can match the time delay between two arms of the interferometer. The light pulse is processed by an EDFA and filter module (EDFA-FT-1550-30-FA-M) with a gain of 23 dBm, bandwidth of 1.6 nm, and center wavelength matched to the laser. The interference pulse is converted by PDs (TCAPDM-150) to a high-speed multi-channel digital acquisition card (NI PXIe-5170R) with a maximum real-time sampling rate of 250 MS/s. Finally, the array signal is processed by the 3 × 3 demodulation algorithm, which can directly obtain the phase changes caused by the acoustic signal.

**Figure 7:**
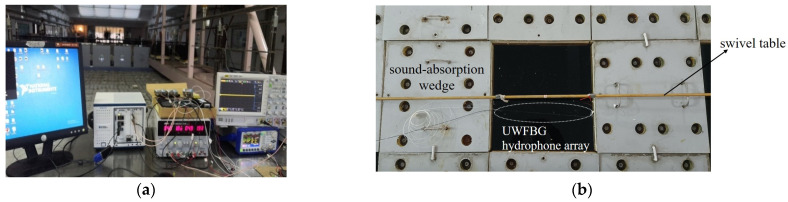
Anechoic pool test site: (**a**) dry ends; (**b**) wet ends.

In the wet ends, the UWFBG hydrophone array attached to the demodulation system is placed 5 m from the acoustic transducer along the anechoic pool so that the acoustic wave generated by the transducer can be transmitted directly to the fiber. The acoustic wave is generated by a transducer driven by a signal generator and passing through the power amplifier. To characterize the response of the fiber-optic hydrophone, an acoustic wave is set at a single frequency for a short time (2 ms). A standard piezoelectric (PZT) hydrophone is placed close by for calibration. The received signal is amplified by an 8102A charge amplifier and filter. After that, the sound pressure information is measured on an oscilloscope. In summary, these are the wet ends of the system, while the dry ends, set at the anechoic test site, are shown in Figure 7a.

Figure 7b shows the site photographs of the swivel table positioned on the sound-absorbing wedge for the anechoic pool. As highlighted in yellow in Figure 6, two ropes were used to suspend the hydrophone in the pool water, keeping it in the same direction as the turntable. Whenever we adjust it, the azimuth of the plane wave with the UWFBG array changes accordingly.

### 3.3. Sensitivity Analysis

**Figure 8:**
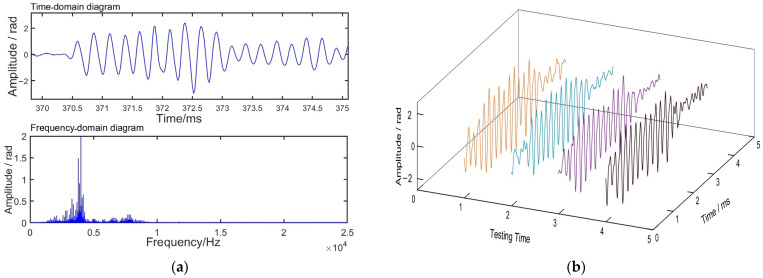
Signals demodulated by FOH: (**a**) signal of phase difference variation; (**b**) amplitude of FOH response by multiple tests.

The acoustic pressure sensitivity is calibrated by the free-field reciprocal method in the experiment. The standard hydrophone signal is used to relate the phase difference to the sound pressure. The sound pressure amplitudes *e_h_* are measured by the PZT hydrophone, which is amplified by 50 dB before the oscilloscope. Assuming that the phase pressure sensitivity of the PZT hydrophone is *M_h_*, in this experiment it can be considered as *M_h_* + 50. Thus, the phase pressure sensitivity of our system is calculated as

(12) $$M=20\mathrm{lg}\frac{e_{\phi }}{e_{h}}+M_{h}+50$$

where the phase changes *e_φ_* are demodulated by array signal processing with identical UWFBGs. It is clear that the sensitivity is closely related to the demodulation amplitude from Equation (12). Figure 8 shows the signals demodulated by optical fiber sensing system.

Figure 8 shows the demodulation results of the hydroacoustic signals @ 4 kHz. In the time domain, it shows that the experimental scheme can be used to demodulate hydroacoustic signals. In the frequency domain, the amplitude spectrum of the demodulation results shows the hydroacoustic signals. Taking the same test condition, the amplitude of the FOH response is stable, as shown in Figure 8b. As the amplitude varies with the direction of the sound wave, it is spatially directive.

**Figure 9:**
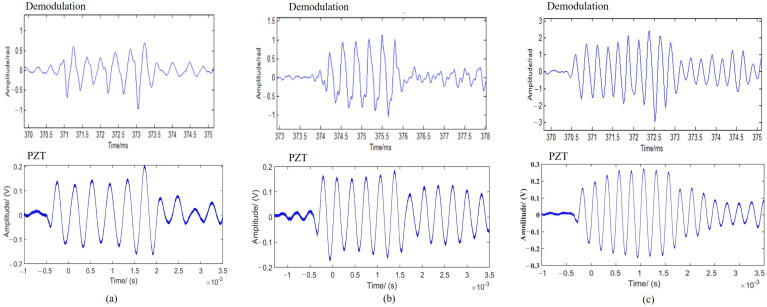
The time response of the hydrophones: (**a**) 2.5 kHz; (**b**) 3.125 kHz; (**c**) 4 kHz.

To test the results of the demodulation with different frequencies of acoustic signals, Figure 9 shows the recorded time response of the FOH and the PZT hydrophone to such a pressure pulse.

Since the acoustic wave is set at a single frequency for a short time (2 ms), as can be seen from Figure 9, the FOH and PZT hydrophones boths have a sinusoidal response as the acoustic wave hits the sensor. However, there is a slight fluctuation at the FOH, which is accompanied by high-frequency noise and is clearly shown in 2.5 kHz and 3.125 kHz. In the anechoic pool experiment, the array is arranged by fiber-optic rings that cannot be straightened. This manual winding process has a much greater effect on the interfering phase signal.

We take the acoustic signal of 4 kHz driven by the transmitting transducer as an example. By Equation (12), the phase pressure sensitivity of our system can be calculated as shown in Table 1. The average phase pressure sensitivity of the array is −140.7 dB (re rad/μPa)@4 kHz.

To achieve spatial sampling and avoid azimuth ambiguity, the spacing between the adjacent fiber rings is normally less than half the wavelength of the sound wave. Given the environmental conditions of the anechoic pool, the frequency response from 2.5 kHz to 6.3 kHz is calibrated in the same way.

**Figure 10:**
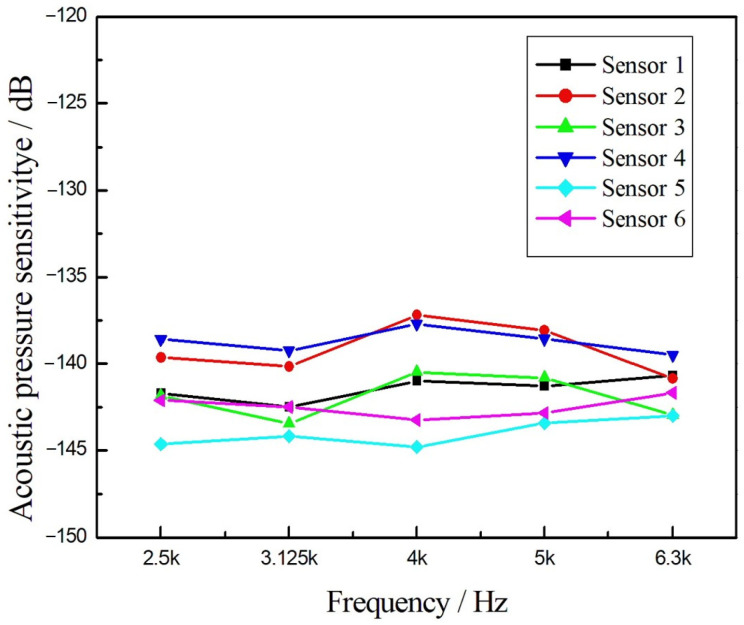
Frequency responses curves of each hydrophone in the six-element array.

The results presented in Figure 10 demonstrate an average acoustic pressure sensitivity of −141.2 dB (re rad/μPa) with a fluctuation of about ±3.5 dB from 2.5 kHz to 6.3 kHz. The reason might be the inconsistency of the fiber length manually wound into the fiber rings. Moreover, it is noteworthy that although the sound pressure sensitivity of a single hydrophone element varies ±1.5 dB from 2.5 kHz to 6.3 kHz, the response is relatively flat.

### 3.4. Azimuth Detection Results

**Figure 11:**
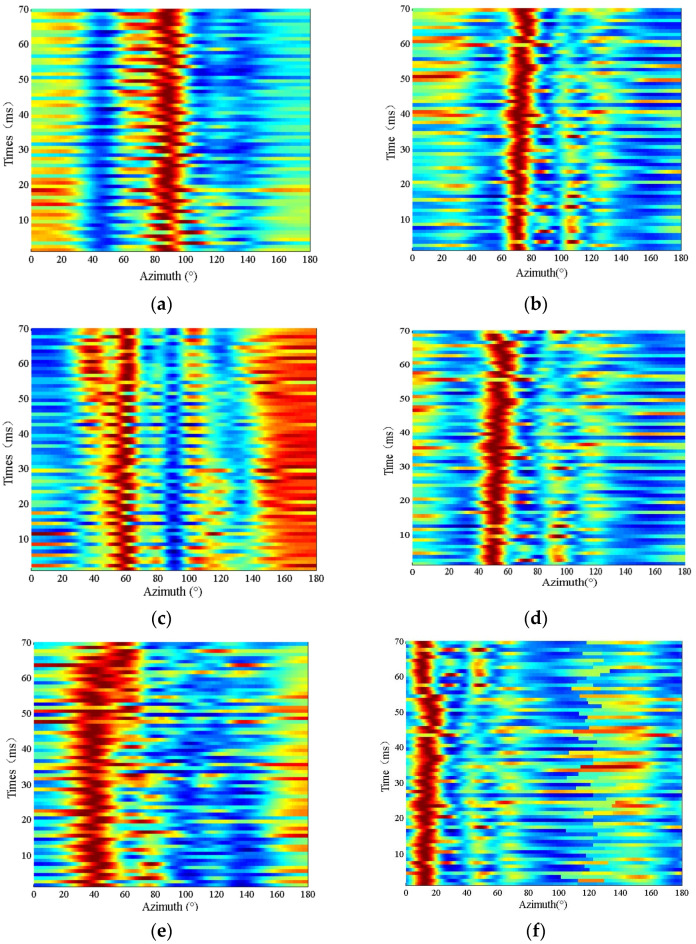
Time–azimuth diagram: (**a**) 90°; (**b**) 75°; (**c**) 60°; (**d**) 45°; (**e**) 30°; (**f**) 15°.

Based on the previous theoretical analysis, the phase difference of the sensor can reflect the target azimuth. We measured the azimuths for a small range of angles due to the limitations of the pool location and the poor quality of the swivel table. In the experiment, we have changed the angle by adjusting the turntable from 90° to 0° at 15° intervals; the results are shown in Figure 11.

In the results, the maximum beam response corresponds to the same azimuth as the acoustic DOA, but fluctuates over short periods. Due to the light weight of the array, there is a deviation of 5°~10° between the array position and the values of each rotation angle. Therefore, this experiment does not test the directional detection accuracy of the array, but the UWFBG hydrophone towed array responds to the acoustic azimuth.

## 4. Conclusions

This paper proposes the millimeter-level FOH towed array based on UWFBG for underwater acoustic direction finding. The experimental results show that this UWBG hydrophone towed array without a substrate winding structure can detect acoustic signals and obtain information such as phase, amplitude, sensitivity, and acoustic direction. The test array is a selection of six sensing fibers, each of which is coiled into a 9 cm diameter fiber ring suspended in the water to receive acoustic signals. An average sensitivity of −141.2 dB re rad/μPa at frequencies from 2.5 kHz to 6.3 kHz was achieved, validating the detection of the azimuth of underwater acoustic waves. The ultra-thin towing cable system, with free structure, high sensitivity and underwater target-detection capability that it has demonstrated has great potential for future unmanned underwater vehicle (UUV) applications.

In addition, this paper has conducted experiments on the direction detection of the UWBG hydrophone towed array in an anechoic pool. Only a six-element FOH array was tested due to the limited space. Although manual winding of the fiber rings has reduced the spacing, it was so difficult to maintain uniformity that noise was introduced. In the future, we will straighten the linear array and place it in an open lake, then optimize the turntable design using a high-power, low-frequency acoustic transmitter to more realistically verify the performance of the distributed UWFBG hydrophone towed array.

## Figures and Tables

**Table 1:** Phase acoustic pressure sensitivity of different sensors.

Hydrophone	*e_φ_* (rad)	*e_h_* (V)	Sensitivity (dB)
S_1_	2.573	0.513	−140.9
S_2_	3.989	0.513	−137.2
S_3_	2.73	0.513	−140.5
S_4_	3.758	0.513	−137.7
S_5_	1.658	0.513	−144.8
S_6_	1.984	0.513	−143.3

## Data Availability

The data presented in this study are not readily available because the data are part of an ongoing project.
